# Artificial intelligence in the medical profession: ready or not, here AI comes

**DOI:** 10.1016/j.clinsp.2022.100010

**Published:** 2022-02-14

**Authors:** Aidan Pucchio, Joshua Del Papa, Fabio Ynoe de Moraes

**Affiliations:** a School of Medicine, Queen's University, Kingston, Ontario, Canada; b Department of Oncology, Queen's University, Kingston, Ontario, Canada; c Kingston Health Sciences Centre, Kingston, Ontario, Canada

## Background

A historical perspective is helpful in understanding the context in which Artificial Intelligence (AI) currently fits within healthcare. When large-scale, systemic leaps in technology take place, rarely are those who stand to benefit from their implementation prepared. Poor preparation leads to widespread process inefficiency, a lack of stakeholder education in the use of the technology, and, at worst, resistance to implementation. The field of medicine is particularly guilty of inefficient adaptation.^1^ Whether for reasons of patient safety, strong traditions of practice, or fear of the dehumanization of medicine, the healthcare system have a long history of inefficient technological uptake.

Uptake of the infamous Electronic Medical Record (EMR) represents a recent example of this phenomenon. EMR technology has the capacity to streamline incredibly time-consuming daily tasks for highly trained physicians, yet widespread implementation has been met with significant delays. The EMR was first introduced in the 1960s, yet over 60 years later, many institutions still cling to antiquated, paper-based charting methods. Even institutions that have adopted the technology frequently run into cross-application compatibility issues and poor technical understanding.^2^ Importantly, widespread EMR adoption is not related to the efficacy of the technology but rather its the thoughtful implementation in clinical settings.

As Artificial Intelligence (AI) tools progress towards clinical implementation, barriers to effective use will be analogous or more significant than those of EMR. AI has already revolutionized other industries; healthcare is not far behind, with AI tools being able to accurately perform tasks such as radiological detection of lung nodules in imaging and other applications already approved for clinical use.^3^ The medical community must engage in active preparation and thoughtful implementation of AI technologies in the clinic, which can only be done through proactive measures in medical education and policy. Here, the authors explore the need for increased interdisciplinary collaboration, financial support, and adaptation in medical education to support the growth and safe implementation of AI in medicine.

## The problems

As healthcare moves to a more digital environment and medical AI tools continue to develop, the knowledge gap between scientists, engineers and clinicians continue to grow.^3^^,^^4^ Physicians tend to have limited knowledge of basic statistics, much less of big data techniques and artificial intelligence.^3^ This can lead to challenges in effective AI implementation, as the individuals developing AI tools are often not aware of the context of tool deployment or their clinical utility, while the physician tasked with implementing AI tools may not understand how the tools work or the role for which they were designed.^3^^,^^4^ This separation in knowledge represents a significant barrier in medical AI research.^3^

This knowledge gap can be ameliorated by a multidisciplinary team, as is illustrated in the work of the Icahn Institute for Genomics and Multiscale Biology at Mount Sinai Health System in New York. Here over 300 staff with diverse backgrounds, including data scientists, bioinformatics experts, and medical geneticists, work on big data research and develop predictive models.^5^ This group continues to drive significant advances in many areas of big data research, with projects including a robust model that predicts the onset of a variety of diseases with high accuracy.^5^

Clinical implementation of AI tools is being further stalled by a lack of robust evidence and policy to support use. This issue arises due to a relative paucity of funding in academic medicine when compared to the AI initiatives in other industries. While AI is pervasive in other industries because it drives profit and is integral to business practices, initiating academic AI research is a significant short-term expense, immediate disincentivizing action and concentrating medical AI work at select centers that have prioritized this research.

As AI tools drive profit in many industries, money trickles down to the AI researchers developing the technology. Many academics with AI expertise are being drawn to the industry, attracted by high salaries, more resources, and more lenient working hours.^6^ Private spending in AI research, including in healthcare, is massive, and if academic opportunities and funding aren't at or approaching parity with industry, talent retention in academia will become a challenge. Business and industry were able to look beyond the high initial cost of AI development, and academic medicine must follow. As the private sector attracts AI experts, medical AI development may struggle in both pace and quality.

Finally, the lack of education on AI in medicine represents one of the most challenging systemic issues. Medical education is largely based on the traditional curriculum. Compounding this issue is the limited and sporadic inclusion of comprehensive statistics, data science, or AI curriculum.^3^ In Canada, few institutions have recognized this growing need, and all educational opportunities have taken the form of elective, condensed programs, such as the University of Toronto's one-week AI in Health certificate.^3^ In the United States, educational opportunities are similarly limited, with significant variance in the content or opportunities offered.^7^

## Solutions

While the barriers to uptake of AI in medicine are significant, the successes are too. AI technology is more accurate and accessible than ever, some medical schools have initiated data science-focused undergraduate medical curriculums, and large academic research centers have prioritized multidisciplinary AI research.^3^

The first priority is to support and incentivize multidisciplinary work in AI in medicine. While the investment in AI research seen at the Mount Sinai Health System highlighted above may not be practical in most academic settings, smaller collaborative efforts such as “Hackathons” or increased incorporation of data and computer scientists into traditional medical research teams are. Medical trainees need to be encouraged and exposed to collaborative work at all levels, and academic centers should make efforts to forge relationships between the trainees in allied faculties.

Secondly, academic funding must shift to promote AI research and development, as medicine must urgently work to retain talent. Not only does this include the hiring of multidisciplinary teams, but salaries and grants must grow to rival industries. Further, the challenging lifestyle of academic physicians and researchers should be reassessed and improved to make academic AI research a more attractive career. Professional associations and physician governing bodies must identify AI research as a priority to push institutions to prioritize it in their hiring practices, staff selection, and financial considerations.

Finally, medical education must shift to embrace data science and AI. The traditional curriculum is quickly growing antiquated and is not serving the needs of medical trainees or the patient population. In addition to the considerations of patients, the next generation of physicians must be considered – while AI is unlikely to replace physicians, it is plausible that physicians who use AI will replace those that do not. Further, physicians will soon be under pressure to adopt AI, and assist in the development, improvement, and integration of AI into clinical workflow, a task they will not be prepared for without urgent curricular accommodation.

## Conclusion

There is a growing urgency to support the future of medical AI development. With pressure from industry, a scarcity of interdisciplinary collaboration, and a lack of AI curriculum in medical education, medicine is at risk of inefficient adaptation and delay, which is damaging to both patients and physicians. The inevitable future of clinically deployed AI will happen whether medicine is ready or not – and the transition must occur with thoughtful integration of these principles into existing infrastructure.

## Conflicts of interest

The authors declare no conflicts of interest.
